# The Dimensions API: a domain specific language for scientometrics research

**DOI:** 10.3389/frma.2025.1514938

**Published:** 2025-10-01

**Authors:** Adam Kövári, Michele Pasin, Alexander Meduna

**Affiliations:** ^1^Digital Science, London, United Kingdom; ^2^Department of Information Systems, Faculty of Information Technology, Brno University of Technology, Brno, Czechia

**Keywords:** Dimensions Search Language (DSL), research analytics, data science, domain specific languages, API, data analysis, scientometrics

## Abstract

We describe the Dimensions Search Language (DSL), a domain-specific language for bibliographic and scientometrics analysis. The DSL is the main component of the Dimensions API (version 2.12.0), which provides end-users with a powerful, yet simple-to-learn and use, tool to search, filter, and analyze the Dimensions database using a single entry point and query language. The DSL is the result of an effort to model the way researchers and analysts describe research questions in this domain, as opposed to using established paradigms commonly used by software developers e.g., REST or SOAP. In this article, we describe the API architecture, the DSL main features, and the core data model. We describe how it is used by researchers and analysts in academic and business settings alike to carry out complex research analytics tasks, like calculating the H-index of a researcher or generating a publications' citation network.

## 1 Motivation and significance

The number and size of bibliographic databases and scholarly metadata sources available online have significantly increased in recent years, as various studies reported (Velez-Estevez et al., 2023; Gusenbauer and Haddaway, 2020). The rise of this multiplicity of data sources makes it easier for researchers to carry out complex research analytics studies and, more generally, has enabled the development of disciplines like the science of science (Fortunato et al., 2018), which is primarily based on techniques like bibliometric analysis and science mapping analysis, to reveal new patterns and insights in scientific output.

Dimensions (Hook et al., 2018) is a research analytics platform that provides access to a large interlinked database of research objects (more than 136 million publications, 7 million grants, 154 million patents, 787,000 clinical trials, and so on, as well as hundreds of millions of links between these objects). Dimensions, originally launched in 2018 (Hook et al., 2018), provides access via a web interface through which users with varying degrees of expertise can easily interact with and explore data using a mixture of full-text search and filtering and faceting approaches.

Alongside the launch of Dimensions' web interface in 2018 an API was also released. Rather than just giving users a standard data harvesting and access API, as was the usual state of the art in 2018 in research information, Dimensions took another step by providing the Dimensions Search Language (DSL) to allow more structured searches of the data through the API. There are many advantages to this approach (Sobernig and Strembeck, 2014), that we will explore below. Dimensions also provided bulk data delivery as an option for certain use cases and, two years later in 2020, a further data access methodology would be added to Dimensions in the form of BigQuery on the Google Cloud Platform (Porter and Hook, 2022). While the Dimensions on Google BigQuery approach has advantages over all of the other data delivery mechanisms described above, it does not, to date, have full-text searching capabilities, and hence the API continues to have significant value to a certain class of technical user or app builder. The focus of this paper will be on the structure of the API and the nature of the DSL.

## 2 Software description

The core component of the API is the Dimensions Search Language (DSL), a domain-specific language aimed at facilitating research analytics tasks such as bibliometrics analysis, data retrieval, and aggregations. It is important to distinguish that in this context, DSL refers specifically to the Dimensions Search Language, not to be confused with the general computer science term “domain-specific language” (also abbreviated as DSL). A domain-specific language is a computer language specialized to a particular application domain, in contrast to a general-purpose language, which is broadly applicable across domains. The Dimensions DSL was specifically developed with the needs of bibliometrics and research analytics practitioners in mind.

Unlike many other relevant APIs from the research analytics world (Velez-Estevez et al., 2023), DSL is a single, uniform platform for querying all types of relevant data, including publications, grants, organizations, clinical trials, researchers, patents, policy documents, and exposes relations between related entities, including ability to filter and facet on them.

The primary reason Dimensions has developed its custom DSL is that it's intended to be used by domain experts, and research analysts who do not necessarily possess the ability to write SQL or utilize RESTful or GraphQL queries (Medjaoui et al., 2021; Vijayakumar, 2018; Gough et al., 2021; Biehl, 2015), and who can quickly become familiar with a simple, human-readable domain language. Other reasons are efficiency and compliance. It is much more efficient to perform a single DSL query that performs all joining and transformation on Dimensions servers, close to actual data, and downloads only the necessary result, than downloading huge amounts of disconnected data, only to perform local processing. Compliance can also be enforced much more easily: Dimensions contains significant amounts of data that are provided under license to Digital Science and, as such, it needs to be delivered to end users according to the terms of the Dimensions data license. The API provides an easy mechanism to allow access to Dimensions in a manner where licensing compliance can, to some extent, be built in.

After years of development, the current API version 2.12.0 is a rather mature and stable application that is used by hundreds of customers and serves millions of requests per day. All examples and functionalities described in this paper were tested using API version 2.12.0 with Python 3.9+ and the dimcli library version 1.4.

## 3 Software architecture

At a high level, the Dimensions API is a web service that (a) accepts a DSL query from end users (b) translates the query into a Solr query^1^ and executes them on Solr based servers, and (c) transforms results into the requested structure so that it can be returned to end users. In addition to executing Solr queries, the API also offers various specialized functionalities to expose additional features Dimensions provides e.g., for classifying or annotating text.

### 3.1 DSL as a service

**Figure 1:**
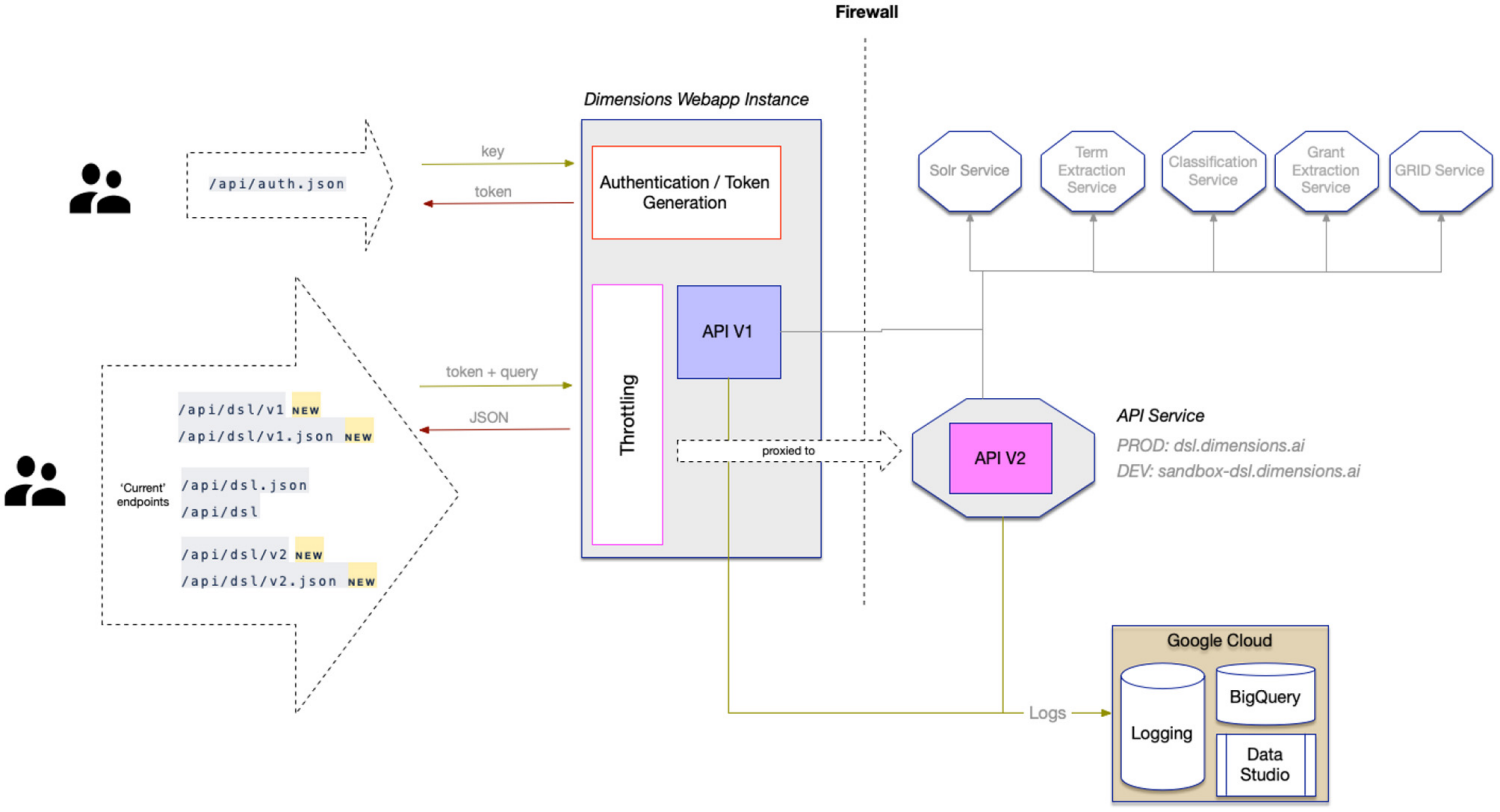
Architecture of the DSL.

The DSL is hosted by a web service that is accessed by various clients, including the Dimensions web application (see Figure 1). The Dimensions website provides a query box, where users can directly type in a DSL query and obtain results in a browser. Clients developing custom integrations with Dimensions data can use a provided web interface. The service itself is deployed in a kubernetes cluster, shielded by a load balancer, which prevents misuse and overloading of the service. Throttling of requests and authentication is performed by the Dimensions.ai site.

The DSL authentication is handled by the /api/auth endpoint, which takes an API key and returns a token to be used when performing DSL queries. Access to the Dimensions API requires a subscription, which provides the necessary API key for authentication. The API key is provided to eligible users via the Dimensions.ai page^2^. The obtained token has a limited validity of one hour and must be renewed thereafter.

The /api/dsl endpoint is used to perform actual DSL queries, while providing the authentication token as a header. The data returned from this endpoint is always JSON format and the request body is just a plain text DSL query.

### 3.2 DSL query execution

The execution of a DSL query consists of the following steps:

1. Parsing and translation of the DSL query to an intermediate representation.
2. Translation from the intermediate representation to a Solr query (queries) (in case of special function it would translate to REST requests to appropriate services).
3. Execution of Solr query (queries).
4. Extraction and reformatting of relevant information from Solr response to the DSL-defined result format.

Different challenges occur in these steps. The query parser is generated using Antlr (Parr, 2012), and the translator uses built-in mappings and configurations to interpret correct source and field information to build the intermediate representation which contains all the necessary data to build a final Solr query, such as information about which fields to return and what filters to apply, what facets to compute, etc. The intermediate representation is then validated against configured mappings and configuration, authorizations calculated, optimized, and only then translated to the final representation.

The translation from the intermediate representation then transforms this information into a Solr request, potentially multiple, if nested entity filters are present. In the case that nested entity filters are present in the query, such as filters that make use of a relation based on content that is held in a different Solr index, more than one Solr query will be needed. Nested entities allow for similar functionality as joins in the world of SQL. They possess certain limitations due to their implementation, such as that they are only possible for IDs of objects, and there is a limit to how many returned IDs can be used in the main query. These restrictions are a cost of the fact that Solr itself has no support for these kinds of queries and it has to be implemented on top of regular queries, and without them, the DSL query execution might be too unstable to bear.

Execution of Solr queries involves executing HTTP requests, with special attributes to prevent overlong queries and then error-catching situations, such as a timeout being exceeded and/or there is some kind of runtime error coming from Solr. Note that at this point, requests to Solr are already validated by the DSL, so in theory they should not happen. Evaluation type of errors are not very common and happen typically for very large result datasets in very specific cases, such as receiving many publications with an extreme set of author affiliations.

The extraction of results from a Solr response consists of mapping individual records using the DSL configuration to the appropriate fields, extracting related and expanded entities, and mapping these additional fields into the requested data shape. Additionally, there are occasionally some post-processing actions defined for several fields, and this is the point at which such post-processing happens.

## 4 Software functionalities

This section describes the functionality of the DSL language.

### 4.1 Data model

In the DSL world, each Dimensions content type is defined as a *source* that has *fields* and field *value types*. Such fields may be used as *filters*—e.g., to limit the results of a query—or as a *facet*—e.g., to aggregate the results of a query. Additionally, the language has *indicators* that are predefined aggregations that can be carried out with facets, and *search indices* that are specialized fields that can be used for advanced full-text searches.

Twelve primary sources can be queried via the API, each corresponding to a collection of documents indexed in Dimensions (Resources, 2023):

1. **Publications:** journal articles/preprints/edited books/book chapters/monographs relating to research, indexed via Crossref, PubMed, PubMed Central, arXiv.org, and more than 160 publishers directly.
2. **Source titles:** a database of publications “containers,” for example, journals, preprint servers, book series, and others.
3. **Datasets:** stand-alone data sets and those associated with publications, from a variety of data repositories indexed via DataCite and Figshare.
4. **Grants:** awarded grants for research projects, coming from hundreds of different funders from across the globe.
5. **Patents:** patent records from IFI Claims from more than 100 countries and patent offices.
6. **Clinical trials:** clinical trial records currently coming from about a dozen registries such as ClinicalTrials.gov (United States), CHICTR (China), UMIN-CTR (Japan), and EU-CTR (European Union).
7. **Policy documents:** government guidelines, reports, or white papers; publications by independent policy institutes, advisory committees on specific topics, research institutes, and international organizations.
8. **Reports:** “gray” literature such as working papers and reports from various different organizations.
9. **Researchers:** disambiguated researchers' profiles extracted from all the primary document types indexed in Dimensions.
10. **Organizations:** disambiguated researcher organizations profiles extracted from all the primary document types indexed in Dimensions.
11. **Funder groups:** curated lists of funders grouped by specific criteria.
12. **Research org groups:** curated lists of research organizations grouped by specific criteria.

Several auxiliary entities exist that cannot be queried directly but are available as structured fields or facets that may be attached to the primary sources listed above, for example, *Repositories, Countries, Cities, States, Categories, Journals, Open Access*, and *Publication Links*.

#### 4.1.1 Cross links

**Figure 2:**
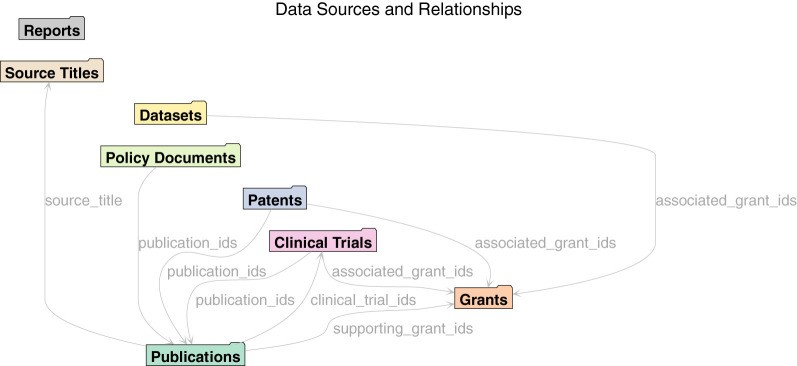
Sources.

**Figure 3:**
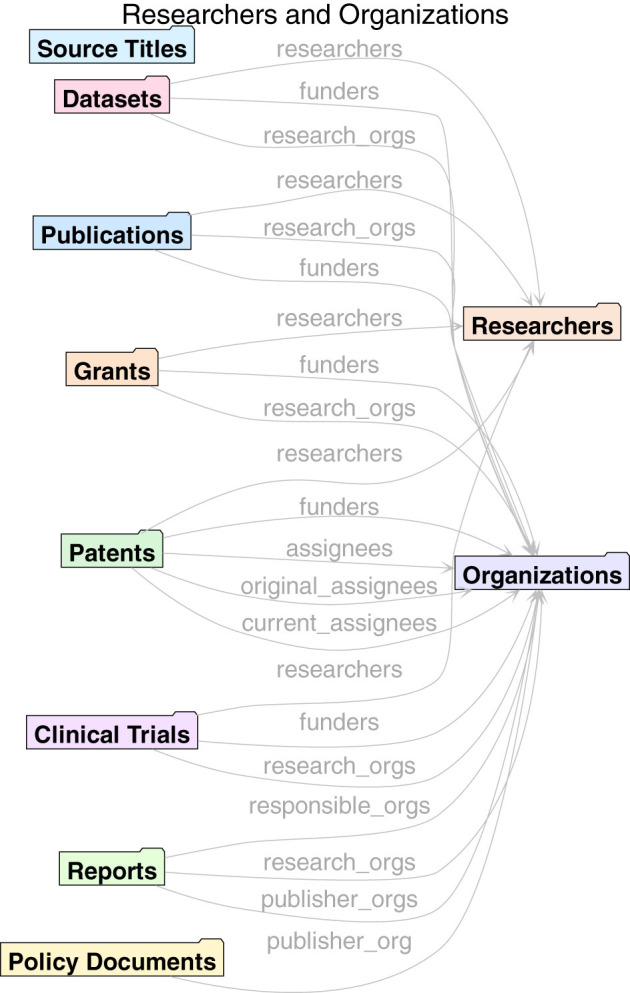
Researchers and organizations.

An advantage of the single unified model behind Dimensions data is that it makes it easier to expose cross-links between entities. There are hundreds of millions of such links between Dimensions sources. Cross-links enable more advanced scientometrics analyses of the data, ranging from traditional citation-based publications analyses to analyses that use links between funding and publications, or between patents and clinical trials and publications. In Figures 2, 3 it is possible to see an overview of the primary cross-links fields available via the API.

#### 4.1.2 Identifiers

Persistent identifiers are foundational elements in the overall research information infrastructure, as they make it easier for research to be shared, validated, and (re)used (Meadows et al., 2019). All entities in the DSL include a unique Dimensions persistent identifier. Whenever possible, external identifiers are added so to make it easier to perform data integrations with other applications and data providers. Table 1 lists external identifiers available in Dimensions data sources.

**Table 1:** Identifiers.

**Source**	**Field**	**Description**
Publications	id	Dimensions ID
Publications	altmetric_id	Altmetric publication ID
Publications	arxiv_id	arXiv ID
Publications	book_doi	The DOI of the book a chapter belongs to
Publications	doi	DOI
Publications	isbn	ISBN
Publications	issn	ISSNs, including both print and electronic
Publications	pmcid	PubMed Central ID
Publications	pmid	PubMed ID
Grants	id	Dimensions ID
Grants	project_number	Grant identifiers, as provided by the source (e.g., funder, aggregator) the grant was derived from
Patents	id	Dimensions ID
Patents	application_number	Number assigned to a patent application when it is filed
Patents	family_id	Identifier of the corresponding EPO patent family.
Clinical trials	id	Dimensions ID
Datasets	id	Dimensions ID
Datasets	doi	DOI
Policy documents	id	Dimensions ID
Source titles	id	Dimensions ID
Source titles	issn	ISSNs, including both print and electronic
Reports	id	Dimensions ID
Reports	doi	DOI
Reports	external_ids	External identifiers available from the report publisher (e.g., OSTI ID)
Researchers	id	Dimensions ID
Researchers	nih_ppid	PI Profile ID from the US National Institute of Health (NIH)
Researchers	orcid_id	ORCID ID
Organizations	id	Dimensions ID
Organizations	cnrs_ids	CNRS IDs
Organizations	external_ids_fundref	Fundref IDs
Organizations	hesa_ids	HESA IDs
Organizations	isni_ids	ISNI IDs
Organizations	orgref_ids	OrgRef IDs
Organizations	ror_ids	ROR IDs
Organizations	ucas_ids	UCAS IDs
Organizations	ukprn_ids	UKPRN IDs
Organizations	wikidata_ids	WikiData IDs

#### 4.1.3 Research categories

Dimensions offer a series of in-built categorization systems that are used by funders and researchers around the world, and which were originally defined by subject matter experts outside of Dimensions (Resources, 2023). Categories are applied at the document level using machine learning techniques that are periodically improved and updated. The API exposes each research category via a dedicated field, for the sources where the category is available. Table 2 lists available categorization systems available in Dimensions data types.

**Table 2:** Categories.

	**Publications**	**Grants**	**Patents**	**Clinical trials**	**Datasets**	**Policy documents**	**Reports**
Fields of research (FOR)	✓	✓	✓	✓	✓	✓	✓
Research, condition, and disease categorization (RCDC)	✓	✓	✓	✓	✓	✓	✓
Health research classification system health categories (HRCS_HC)	✓	✓	✓	✓	✓	✓	✓
Health research areas (HRA)	✓	✓	✓	✓	✓	✓	✓
Broad research areas (BRA)	✓	✓	✓	✓	✓	✓	✓
ICRP common scientific outline (ICRP_CSO)	✓	✓	✓	✓	✓	✓	✓
ICRP cancer types (ICRP_CT)	✓	✓	✓		✓	✓	✓
Units of assessment (UOA)	✓	✓					✓
Sustainable development goals (SDG)	✓	✓			✓		✓
Mesh	✓			✓			
Cooperative patent classification categorization (CPC)			✓				
International patent classification reform categorization (IPCR)			✓				

### 4.2 Query language

The DSL language provides multiple types of queries:

- Full text/filter query: used to search & filter on source records, such as patent applications, or publications; returning a set of matching records.
- Faceting query: can be used in combination with full text/filter queries, but the returned data set is aggregated rather than matching queries, for example obtaining some published articles by a certain organization, grouped by a year of publication.
- Expert identification query: complex query that accepts concepts (which can be extracted from an abstract by the DSL itself too), can contain additional filters, and produces a set of most relevant researchers matching selected concepts and filters, potentially annotated by an organizational and co-authorship overlap.
- Special function calls: these additional function expressions can be used to invoke supporting functionality, such as concept/grant/affiliation extraction or abstract classification.
- Reflection query (describe): metadata about the supported sources, entities, and fields.

Further sections will explore individual types of queries in greater detail.

#### 4.2.1 Full-text, filter, facet query features

This type of query in its basic form contains a type of data source to query and fields to return. This type of query starts with the search keyword and is followed by a full-text query and filter query part. For example:

search publications for “CRISPR/CAS9”

where year in [2013:2018] and type=

“article”

return publications

  [title+authors+doi+year+journal+times_

      cited]

  sort by times_cited

return research_orgs

return funders

This query contains the following items (the only required item is the source dataset):

- Source dataset to query, i.e., *publications, researchers, grants*, etc.
- Specification of a full-text search phrase in a Lucene format, in a specified search index. Boolean operators, wildcards, and proximity search are supported on supported search indexes.
- Filters can be specified as well, these can be viewed as “where” filters in SQL. They can be combined using usual boolean operators. Nested fields, such as publications researchers can be queried as well, using a dot notation, e.g., where researchers.id = ‘‘...''.
- There is also advanced support for searching using researchers, authors, and investigators' names.
- Special support for searching and filtering using concepts, depending on the complexity of a query allows for either basic filtering, or using fully powered Lucene-based search indexes with concepts.
- Support for wildcards (*, ?) and proximity searches ( ) in text queries.
- Specification of a result requires a selection of fields and/or fieldsets to return. In the case of facet queries, facet specification includes also a selection of fields/fieldsets to return, together with the specification of an aggregate operation.
- Sorting, page size, offset to allow obtaining additional data, if necessary.
- Advanced result function expressions allow calculating citations per year and funding per year in a very convenient manner.

#### 4.2.2 Expert identification query

This query allows users to quickly identify suitable experts to review a certain paper. This query performs a proprietary analysis aimed at identifying the most relevant experts in the field defined by a set of key concepts. For example:

identify experts

  from concepts

  ‘‘+malaria OR \‘effective malaria vaccine

   \'''

using publications

where research_org_countries is not empty

  and year >= 2013

return experts[basics]

limit 20 skip 0

The actual execution of this query is in two steps. The first one is to extract key concepts from a paper abstract. Then the actual “identify experts” query continues, specifying identified concepts, and additional filters, overlap checks, and return object specification (fields and fieldsets of identified researchers to return).

#### 4.2.3 Special function calls

Function calls are a special type of query to retrieve different types of extractions, such as grant, concepts, and affiliations extraction. These are helper functions, provided for convenience to Dimensions users, and can be effectively used in combination with other functionality, such as expert identification. In addition to these special functions, DSL also provides a metadata interface that can be used to query information about the structure of the language itself, such as what fields, and entities are supported, etc.

## 5 Illustrative examples

Although the DSL by itself can deliver insightful analytics, for some more advanced use cases it is necessary to complement it with an external programming language to perform loops and additional data processing. In this section we provide examples of various applications, from business intelligence to complex multi-source queries and bibliometric calculations.

### 5.1 Patent landscape analysis

This example shows how pharmaceutical companies may use it to analyze patent landscapes and identify emerging research trends. The following query demonstrates how to identify organizations filing patents related to CRISPR technology and their associated publications:

search patents

  in title_abstract_claims for

    ‘‘CRISPR Cas9"

where year >= 2018

return patents[id+publication_ids]

return assignees[name]

 aggregate count sort by count limit 20

This query returns the top 20 patent assignees in CRISPR technology along with their publication output, enabling companies to identify key competitors and potential collaboration partners. By cross-linking patents with publications and grants data, businesses can assess the research pipeline and make informed decisions about R&D investments.

### 5.2 Research impact assessment

The following example demonstrates the DSL's capability to perform complex scientometric analyses by linking publications to their associated research outputs. This query identifies highly-cited AI research and traces its funding sources:

search publications

   in title_only for ‘‘artificial

   intelligence''

where year>=2020

 and times_cited>50

return publications[doi+title+times_cited+

   authors+supporting_grant_ids]

 sort by times_cited limit 100

return researchers[id+first_name+last_name+

     current_research_org]

This integrated analysis reveals the relationship between high-impact AI publications, their funding sources, and the researchers involved. By linking these data types, institutions and funders can assess research ROI and identify successful funding patterns that lead to highly-cited outputs.

### 5.3 Calculating the H-index

Listing 1 shows how to calculate H-index^3^ using the DSL via the Python library dimcli^4^. The h-index is an author-level metric that attempts to measure both the productivity and citation impact of the publications of a scientist or scholar (Hirsch, 2005). The index is based on the set of the scientist's most cited papers and the number of citations that they have received in other publications. The h-index is defined as the maximum value of h such that the given author/journal has published h papers that have each been cited at least h times. These are the steps we follow:

1. We take a researcher ID e.g., ur.01357111535.49 and save its ID into a variable that can be referenced later.
2. The h-Index function takes a list of citations and outputs the h-index value as explained above.
3. To pass some real-world data to the H-Index function, we use the Dimensions API to extract all publication citations for a researcher, via the get_pubs_citations function.
4. Finally, we combine the two functions to calculate the H-Index for a specific researcher.

**Listing 1:** H-index calculation

import dimcli
dimcli.login()
dsl = dimcli.Dsl()
RESEARCHER = ‘‘ur.01357111535.49''
def the_H_function(sorted_citations, n = 1):
"""
from a list of integers [n1, n2 ..]
representing publications citations,
return the max list-position which is
>= integer
e.g.,
>>> the_H_function([10, 8, 5, 4, 3]) < = 4
>>> the_H_function([25, 8, 5, 3, 3]) < = 3
>>> the_H_function([1000, 20]) < = 2
"""
if sorted_citations and sorted_citations
[0] >= n:
return the_H_function(sorted_citations
[1:], n+1)
else:
return n-1
def get_pubs_citations(researcher_id):
q = """
search publications where researchers.id
= ‘‘{}''
return publications[times_cited]
sort by times_cited limit 1000
"""
pubs = dsl.query(q.format(researcher_id))
return pubs.as_dataframe().fillna(0)
[‘times_cited']
print(‘‘H_index is:'',
the_H_function(get_pubs_citations
(RESEARCHER)))

### 5.4 Citation network analysis

Beyond individual metrics, the DSL enables construction of citation networks to analyze research influence patterns. The following example demonstrates how to build a citation network for a specific research topic:

search publications

in title_abstract for ‘‘CRISPR genome

editing''

where year in [2018:2023]

and type = ‘‘article''

return publications[id+doi+title+reference_

ids+times_cited]

limit 500 sort by times_cited

This query retrieves publications with their reference lists, enabling network construction. Using the dimcli library, researchers can then process this data to create citation graphs using libraries like NetworkX (Hagberg et al., 2008):

import dimcli

import networkx as nx

# Execute query and build network

results = dsl.query (query_string)

pubs_df = results.as_dataframe()

# Create directed graph using networkx library

G = nx.DiGraph()

for idx, pub in pubs_df.iterrows():

pub_id = pub['id']

G.add_node(pub_id, title=pub[‘title'],

citations=pub[‘times_cited'])

# Add edges for references

if pub[‘reference_ids']:

for ref_id in pub[‘reference_ids']:

G.add_edge(pub_id, ref_id)

# Analyze network properties

print (f“Network density: {nx.density(G):.4f}”)

print(f“Average clustering: {nx.average_

clustering(G):.4f}”)

Such network analyses reveal citation patterns, identify seminal papers through centrality measures, and detect research communities through clustering algorithms. The DSL's ability to retrieve both forward citations (through times_cited) and backward citations (through reference_ids) enables comprehensive bibliometric network studies that would require multiple queries in traditional REST APIs.

### 5.5 Advanced bibliometric indicators

Beyond raw citation counts, the DSL provides multiple sophisticated aggregation indicators for comprehensive research assessment. These include field-normalized metrics such as the Relative Citation Ratio (rcr_avg) and Field Citation Ratio (fcr_gavg), citation-based indicators (citations_avg, citations_median, recent_citations_total), and alternative metrics (altmetric_avg, altmetric_median). The following example demonstrates multi-dimensional research impact assessment:

search publications

where research_org_names=

‘‘Stanford University''

and year in [2018:2023]

and type=‘‘article''

return funders aggregate rcr_avg, fcr_gavg,

altmetric_avg

sort by rcr_avg limit 20

return year aggregate rcr_avg,

recent_citations_total, count

This query reveals which funders support the highest-impact research at Stanford across multiple dimensions—field-normalized citation impact (RCR and FCR), social media attention (Altmetric), and recent citation momentum. The RCR, co-developed with NIH (Hutchins et al., 2016), normalizes citation rates relative to peer publications in the same field and year, with values above 1.0 indicating above-average impact. The availability of these diverse indicators as aggregation functions enables nuanced bibliometric analyses that account for disciplinary differences, temporal dynamics, and broader societal impact beyond traditional citations.

## 6 Impact

Scientometrics constitutes a very dynamic area of research as demonstrated by (Gusenbauer and Haddaway 2020). The Dimensions is pursuing a heavily connected approach connecting all relevant research areas such as publications, patents, funding data, datasets, and others. The DSL is a further extension to an already rich ecosystem of Dimensions, but also other commonly used query languages, such as Lucene or SQL. It has provided Dimensions users with advanced analysis capability that can be used to formulate human readable queries searching, filtering, faceting, and connecting the data of Dimensions. The DSL serves dozens of active institutional users, daily serving hundreds of thousands of queries, extracting on average multiple millions of records in total.

## 7 Conclusions

The increasing availability of high-quality databases for scientometrics and bibliometrics analyses calls for more advanced mechanisms to access and query them programmatically. Traditionally, RESTful APIs (Velez-Estevez et al., 2023), have been developed for that purpose, but although these are efficient enough for custom integrations and developers-led projects, they often fall short when it comes to the needs of data analysts, scientometrists and other, not necessarily developer-like audiences. The Dimensions API and Search Language were developed to address this gap. In this paper, we presented the main characteristics of the language, as well as provided examples of its expressivity and power to carry out complex data analytics tasks.

### 7.1 Interoperability and integration

The DSL's comprehensive support for external identifiers (Table 1) plays a crucial role in fostering interoperability between different research information systems. By including identifiers such as DOI, ORCID, ROR, and others, the DSL enables seamless data integration with other platforms and services. This interoperability is essential for creating comprehensive research analytics pipelines that combine data from multiple sources. For instance, researchers can cross-reference Dimensions data with institutional repositories, funder databases, or citation databases to create enriched datasets for analysis. The standardized categorization systems (Table 2) further enhance interoperability by providing common taxonomies that facilitate data comparison and aggregation across different platforms.

### 7.2 Technical Limitations

While the DSL provides powerful capabilities for research analytics, certain technical limitations exist due to the underlying architecture^5^. The API enforces strict limits to ensure performance: a maximum of 1,000 records per single query, 50,000 records total via pagination, and rate limiting of 30 requests per IP address per minute. Query complexity is constrained to 400 items in filter clauses, 100 boolean filter conditions, and 100 boolean full-text search clauses^6^. These restrictions can impact queries involving large-scale cross-referencing between entities. The lack of native support for certain advanced statistical operations means users must export data for external processing when performing sophisticated analyses like network analysis or machine learning tasks.

Although formal comparative benchmarking against other bibliometric APIs was not conducted in this study, the DSL offers distinct qualitative advantages. Its domain-specific syntax significantly reduces query complexity compared to generic REST or GraphQL APIs—tasks requiring multiple endpoint calls and manual data joining in traditional APIs can often be accomplished with a single DSL query. Response times typically remain under 5 seconds for standard queries, with more complex aggregations completing within 30 seconds. Most importantly, the learning curve for non-technical users is substantially reduced; researchers familiar with bibliometric concepts can write effective queries after minimal training, whereas SQL or GraphQL would require extensive programming knowledge.

While the DSL supports multiple field-normalized metrics through aggregation indicators including rcr_avg and fcr_gavg, certain advanced bibliometric calculations remain beyond its current capabilities. Individual publication-level normalized values and percentile-based indicators requiring complete field-specific citation distributions are not directly accessible as raw data, though various aggregate metrics (RCR, FCR, citation statistics, Altmetric scores) can be calculated for groups of publications. These limitations reflect design decisions prioritizing query simplicity and performance over comprehensive statistical functionality, while still providing essential normalized metrics for research assessment.

## Data Availability

The original contributions presented in the study are included in the article/supplementary material, further inquiries can be directed to the corresponding author.

## Footnotes

^1^Apache Solr is an open-source enterprise search platform built on Apache Lucene, providing full-text search, faceted search, and real-time indexing capabilities.

^2^For comprehensive API documentation, see https://docs.dimensions.ai/dsl/.

^3^Digital Science is not a supporter of the use of metrics such as H-Index since it is not aligned with DORA nor is it objectively a good metric - it is highly reductionist and hence treating it and describing it as an example of an “analytics task” (abstract) is not ideal. Other metrics such as RCR (Hutchins et al., 2016) (co-developed with NIH) are generally preferred, however, this section is included to demonstrate the algorithmic approach that could be used to calculate it nonetheless.

^4^The dimcli Python library documentation is available at https://github.com/digital-science/dimcli and can be installed via pip: pip install dimcli.

^5^For detailed technical limits, see https://docs.dimensions.ai/dsl/usagepolicy.html#technical-limits.

^6^Query optimization guidelines available at https://docs.dimensions.ai/dsl/faq.html#are-there-any-guidelines-regarding-query-length-complexity.
